# Blood-Based Lateral-Flow Immunoassays Dipstick Test for Damaged Mitochondrial Electron Transport Chain in Pyruvate Treated Rats with Combined Blast Exposure and Hemorrhagic Shock

**DOI:** 10.3390/jcm14030754

**Published:** 2025-01-24

**Authors:** Evans Okonkwo, Biswajit Saha, Geetaram Sahu, Alakesh Bera, Pushpa Sharma

**Affiliations:** Department of Anesthesiology, Uniformed Services University of the Health Sciences, Bethesda, MD 20814, USA

**Keywords:** blast injury, hemorrhagic shock, sodium pyruvate resuscitation, plasma lactate, mitochondrial complex I, complex IV, PDH, enzyme activity

## Abstract

Blast trauma presents a unique challenge due to its complex mechanism of injury, which impacts the brain and other vital organs through overpressure waves and internal bleeding. Severe blood loss leads to an inadequate oxygen supply and insufficient fuel delivery to cells, impairing ATP production by mitochondria—essential for cell survival. While clinical symptoms of metabolic disruption are evident soon after injury, the molecular, cellular, and systemic damage persists for days to years post-injury. Current challenges in treating traumatic brain injury (TBI) stem from (1) the lack of early blood-based biomarkers for detecting metabolic failure and mitochondrial damage and (2) the limited success of mitochondrial-targeted therapeutic strategies. **Objectives:** To identify blood-based mitochondrial biomarkers for evaluating the severity of brain injuries and to investigate therapeutic strategies targeting mitochondria. **Methods:** A preclinical rat model subjected to blast exposure, with or without hemorrhagic shock (HS), followed by resuscitation was utilized. Blood samples were obtained at baseline (T0), post-injury (T60), and at the conclusion of the experiment (T180), and analyzed using a validated dipstick assay to measure mitochondrial enzyme activity. **Results:** Blast and HS injuries led to a significant decrease in the activity of mitochondrial enzymes, including complex I, complex IV, and the pyruvate dehydrogenase complex (PDH), compared to baseline (*p* < 0.05). Concurrently, blood lactate concentrations were significantly elevated (*p* < 0.001). An inverse correlation was observed between mitochondrial enzyme dysfunction and blood lactate levels (*p* < 0.05). Treatment with sodium pyruvate post-injury restored complex I, complex IV, and PDH activity to near-baseline levels, corrected hyperlactatemia, and reduced reactive oxygen species (ROS) production by mitochondria. **Conclusions:** Serial monitoring of blood mitochondrial enzyme activity, such as complex I, complex IV, and PDH, may serve as a valuable tool for prognostication and guiding the use of mitochondrial-targeted therapies. Additionally, mitochondrial enzyme assays in blood samples can provide insights into the global redox status, potentially paving the way for novel therapeutic interventions in TBI.

## 1. Introduction

Blast injuries resulting from the detonation of improvised explosive devices (IED) in the Iraq and Afghanistan wars caused more military casualties (65.5%) than any other combat-related injury [1,2]. Approximately 20 % of potentially preventable late deaths are caused by traumatic brain injury (TBI), multiorgan dysfunction (MOD), burn and airway obstruction, and hemorrhagic shock/reperfusion (HS/R) [3,4]. Despite the progress in the treatment of blast-injured patients, and the reduced evacuation time, the mortality rate in the battlefield injuries is still high [5,6]. Blast trauma is unique because of its complex mechanism of injury, including that (1) the rapid change in air pressure can cause hemorrhage and perforation in air-filled organs such as the lungs, intestine, ears, and concussion, and (2) the flying objects (debris and bomb fragments) can also cause severe hemorrhage in any part of the body resulting in hemorrhagic shock (HS) [3,7,8]. The role of first responders is to (1) stabilize the patient and (2) prevent the progression of secondary cell injury and multiorgan dysfunction (MOD).

The tissue elements that are not destroyed immediately following a blast and severe hemorrhage may be sub-acutely injured by secondarily generated auto destructive factors such as free radicals, neuroendocrine, metabolic, inflammatory, and transcriptional responses from damaged mitochondria [9,10]. Mitochondrial dysfunctions are mainly caused by the disintegration of the oxidative phosphorylation process and the damaged electron transport chain (ETC) resulting in cellular ATP depletion, increased lactate production by the increased metabolism of glucose through anaerobic respiration, cell death, multiorgan failure, and neurocognitive deficits are just few of them [11,12]. Therefore, early diagnosis of mitochondrial ETC damage and mitochondrial-targeted treatments may save lives by preventing secondary cell injury, organ dysfunction, and cognitive deficits following blast and hemorrhage.

Mitochondria are the powerhouse of the cell, producing ATP by processing glucose-derived pyruvate through the pyruvate dehydrogenase complex (PDH) and facilitating oxidative phosphorylation (OXPHS) via the coordinated functions of the electron transport chain (ETC). The ETC consists of five multi-subunit enzyme complexes: NADH-ubiquinone reductase (complex I), succinate-ubiquinone reductase (complex II), ubiquinone-cytochrome c reductase (complex III), cytochrome c oxidase (complex IV), and ATP synthase (complex V).

The reason behind the development of our blood-based lateral flow immunoassays dipstick test is that mitochondria are present in the mononuclear cells circulating in peripheral blood [13]. Our published study in patients with severe TBI suggest that the enzymes of mitochondrial OXPHOS (pyruvate dehydrogenase complex [PDH], complex I, IV) can be measured in blood by this sensitive blood-based dipstick test [14]. This test works to monitor the enzyme activity of mitochondrial complex I, IV, and PDH in the presence of suitable enzyme substrate, co-factor, and electron acceptor which causes a color change on the dipstick. The intensity of color change indicates the amount of enzyme activity in blood.

Based on the above studies, if mitochondria are one of the key players in the outcome of injured patients with combined blast and HS injuries, then preventing mitochondrial damage by pyruvate administration may be a novel avenue in this study. During OXPHOS, PDC is responsible for providing reducing equivalents from pyruvate to the complex I of the electron transport chain. Pyruvate is a monocarboxylate produced at the end of glycolysis and is the primary source of NADH reducing equivalents for the mitochondria required for OXPHOS to generate ATP. Studies from our laboratory and others have demonstrated the potential of parenteral pyruvate administration in mitigating cell death, minimizing myocardial infarct size, safeguarding organ function, and improving survival rates due to hemorrhagic shock and traumatic brain injury (TBI) [15,16,17]. We have shown that pyruvate improves mitochondrial function and ATP production during hemorrhagic shock, and it is also associated with increased concentrations of reduced glutathione, decreased oxidative stress, decreased apoptosis, decreased poly (ADP-ribose) polymerase-1 activation with higher levels of intracellular NAD, and improved cardiovascular functions [18,19,20]. In this study, we have examined the enzyme activities of PDH, complex I, and complex IV using the dipstick test to assess for mitochondrial damage in the plasma of rats at various time points following exposure to 20 PSI blast alone or combined blast injury and HS, and treated with sodium pyruvate to determine if altered mitochondrial enzyme activities and pyruvate treatment in a rat model of combined blast and HS injury can predict the outcome of animal survival. We have also tried to correlate these mitochondrial enzyme activities with plasma lactate levels, which is an established clinically relevant biomarker of global cellular metabolic status and a predictor of injury outcome.

## 2. Materials and Methods

### 2.1. Ethical Compliance and Animal Study Protocol

This study was conducted in strict compliance with the National Institutes of Health (NIH) guidelines for animal research. Approval for the experimental procedures was obtained from the Institutional Animal Care and Use Committee (IACUC) of the Uniformed Services University of the Health Sciences (USUHS), Bethesda, MD, USA (Protocol ID: ANE-18-045; Date: 8 February 2021).

### 2.2. Study Design and Experimental Groups

The following groups of male Sprague Dawley rats (300–350 g) were utilized for this study:

1. Sham group (n = 8): Rats in this group underwent anesthesia and catheter placement but were not subjected to injuries or resuscitation. Blood samples were collected solely for laboratory analyses.
2. Control groups (n = 16, divided into two subgroups): These animals experienced blast injuries with or without hemorrhagic shock (HS) and received hypertonic saline (HTS) as a resuscitation fluid at a rate of 5 mL/kg/h. HTS was selected to match the volume and osmotic characteristics of the experimental treatment fluid.
3. Treatment groups (n = 16, divided into two subgroups): rats in this group were exposed to the same injury conditions (blast with or without HS) and were resuscitated with sodium pyruvate administered at 5 mL/kg/h.

### 2.3. Preparation of Animals and Surgical Setup

The experimental procedures followed a well-established protocol. Male rats were anesthetized with 5% isoflurane with 100% oxygen using an induction chamber. Maintenance of anesthesia was achieved via a nose cone with 1.5–2% isoflurane with 100% oxygen. To facilitate experimental manipulations, femoral arteries and veins were catheterized with PE50 tubing, secured using surgical sutures.

Monitoring and sampling: A catheter in the right femoral artery was linked to a pressure transducer for continuous monitoring of mean arterial pressure (MAP), while a catheter in the tail artery enabled baseline blood sampling. MAP and heart rate were recorded at 5 min intervals using automated software.

Induction of hemorrhagic shock (HS): controlled blood withdrawal through the left femoral artery induced HS, while fluid or blood infusions were administered via the left femoral vein using an infusion pump.

Temperature regulation: body temperature was maintained within a physiological range (37–37.5 °C) using a rectal probe and an electric heating pad.

### 2.4. Blast Injury

All animals, regardless of whether they underwent shock wave TBI or a sham procedure, were placed under anesthesia using 2–4% isoflurane with 100% oxygen in a transparent induction chamber. The absence of a paw-pinch reflex confirmed the sufficient depth of anesthesia before initiating the procedure. Animals in the blast TBI group were exposed to shock waves generated by the USUHS preclinical behavior and modeling core’s (PBMCs) advanced blast simulator (ABS), [21]. The ABS mimics blast wave exposure, which has been associated with neuropathological changes [22,23]. Blast overpressure (BOP) exposure, simulated in a laboratory shock tube by rupturing Mylar membranes at specific pressure thresholds, can lead to both structural and functional alterations in the brain [24]. Rats were individually secured in a holder that is inserted inside the distal end of the shock tube. After sustaining a short duration shock wave (<21 msec, peak pressure 20 psi), the rat was immediately removed from the ABS and placed back into the anesthesia chamber to remain anesthetized. The ABS was prepared for a second shock wave, and the rat was placed back in the ABS. This procedure was followed a total of three times with repeat shockwave exposure at an interval of 15 min. The rats were subsequently returned to their home cages, kept on a warmer set to 38–40 °C, and observed until they regained consciousness, indicated by the righting reflex. In contrast, animals in the sham group were anesthetized and positioned in the holder at the distal end of the shock tube without exposure to shock waves. Previous observations in the lab suggest brief isoflurane and ABS exposure had no adverse behavioral effects.

### 2.5. Induction of Controlled Hemorrhage and Resuscitation Procedures

Controlled hemorrhage protocol (T0–60): Rats were allowed a 5 min acclimation period before initiating experimental procedures, marked as time zero (T0). Baseline circulatory variables, including mean arterial pressure (MAP) and pulse rate, were recorded. Controlled blood withdrawal was performed over a 15 min span to achieve a target MAP of 40 mmHg. Blood samples were collected in pre-heparinized tubes. MAP was maintained at 40 mmHg for 40 min through regulated withdrawal or infusion of the shed blood as needed.

Fluid resuscitation and blood reinfusion (T60–150): Following hemorrhagic shock (HS), animals received resuscitation with either hypertonic sodium pyruvate (2 M) or hypertonic saline (7.5%) at a rate of 5 mL/kg/h from T60 to T120. Subsequently, the collected blood was reinfused into the animals during the T120–T150 period to restore circulatory stability.

## 3. Analytic Methods

### 3.1. Blood-Based Dipstick Test for the Measurement of Mitochondrial Enzymes as Biomarkers of Mitochondrial Damage

Blood samples collected at baseline (T0), post-HS (T60), and at the end of the experiment (T180) were analyzed for mitochondrial enzyme activities, including complex I, complex IV, and pyruvate dehydrogenase complex (PDH). The dipstick assay, developed based on immunologic sandwich techniques, followed manufacturer guidelines (Abcam, Cambridge, UK) and prior validated methods. The intensity of the color bands on the dipsticks was measured using a flatbed scanner, and data were analyzed with ImageJ software. This assay was preferred over conventional spectrophotometric methods due to its quicker turnaround time (about 45–50 min), the elimination of mitochondrial isolation, and its cost-effectiveness for smaller sample sizes with greater sensitivity. The technique is very sensitive with the small size of the analytical kit, higher sensitivity to a smaller amount of target proteins (15–25 μL blood), and being more cost-effective because each dipstick costs about $7–10 [14,25].

Measurement of lactate levels: Lactate concentrations in whole blood were quantified using a handheld i-STAT device (Abbott Laboratories, Santa Clara, CA, USA) equipped with CG4+ cartridges. This enabled rapid evaluation of respiratory status and detection of hyperlactatemia with minimal blood volume (80 μL).

### 3.2. Statistical Evaluations

Group sizes were determined through power analysis using SigmaStat v4.0 software, guided by prior published studies. Data concerning mitochondrial ETC complex activities (I and IV) and PDH in response to blast and HS, with or without pyruvate treatment, were analyzed using one-way ANOVA followed by LSD multiple comparisons. A *p*-value of <0.05 was deemed statistically significant.

## 4. Results

### 4.1. Sodium Pyruvate Resuscitation Improves Mean Arterial Pressure in Response to Blast and/or HS Injury

**Figure 1:**
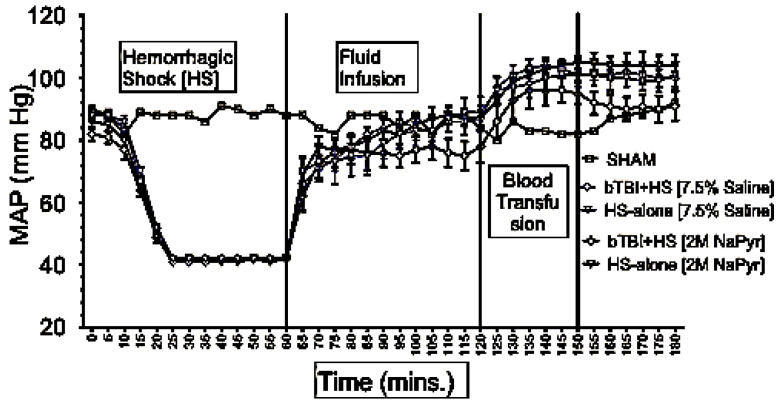
Effect of sodium pyruvate infusion on mean arterial pressure (MAP) in rats with combined blast injury and HS. Data represents mean ± SE of 8 animals in each group. All rodents had similar MAP at the baseline level and HS periods of the experiment. During the HS, the MAP was maintained within 40 s as intended. Both fluids increased MAP towards the baseline after infusion.

**Figure 2:**
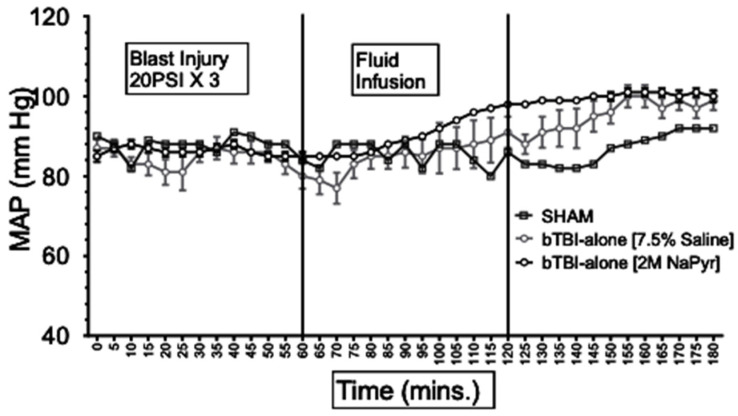
Effect of sodium pyruvate infusion on mean arterial pressure (MAP) in rats with blast injury alone. Data represents mean ± SE of 8 animals in each group. All rodents had similar MAP at the baseline level and HS periods of the experiments. During the HS, the MAP was maintained within 40 s as intended. Both fluids increased MAP towards the baseline after infusion.

Since organ specific blood flow in response to injury and/or blood loss cannot be directly measured in clinical settings, mean arterial blood pressure (MAP) is commonly used as an estimate of tissue perfusion. Data depicted in Figure 1 show that MAP was similar in all animals at the baseline (85–90 mmHg) and decreased to approximately 40 mmHg in all HS animals during the first 15–20 min period of blood loss. The HS was maintained for a total duration of 60 min. Because of similar weights of the rats, the total shed blood volume did not differ significantly among the different rats (8.42 ± 0.15 mL of blood loss). Mean arterial pressure was measured at 0–180 min in all animals, and differences were noted among the different injury and treatment groups as follows: (i) sham animals, the MAP of the sham group receiving no treatments remained stable between 80 and 92 mmHg throughout the experiment; (ii) sodium pyruvate or hypertonic saline infusion in the injured animals (T60–120), both pyruvate and hypertonic saline (7.5% saline) infusions were equally effective in increasing the MAP to, or near, the baseline values after the HS+/−bTBI (*p* < 0.0001). During the pyruvate resuscitation, MAP reached a maximum of 78 ± 5 mmHg in the blast + HS group and 88 ± 3 mmHg in the HS-alone group. Similarly, HTS infusion increased the MAP to the maximum of 87 ± 5 mmHg in the blast + HS animals and 89 ± 5 mmHg in the HS-alone animals. It was noticeable that in comparison to the single injury group with HS alone, the combined injury groups of bTBI and HS had less increase in MAP during the resuscitative period with sodium pyruvate or hypertonic saline solution. Blood transfusion was effective in stabilizing the MAP above baseline till end in all HS+/−bTBI injury and treatment groups. Following both pyruvate and blood infusions, MAP overshot the baseline to 96 ± 5 mmHg in the blast + HS group and 105 ± 3 mmHg in the HS-alone group. Similarly, MAP reached the maximum of 104 ± 3 mmHg in the blast + HS animals and 101 ± 4 mmHg in the HS-alone group after both HTS and blood infusions. Data depicted in Figure 2 show that the MAP of the bTBI (20PSI)-only animals remained stable throughout the experiment.

The adequate tissue perfusion or flow of blood through tissues regulated by MAP is essential for delivering oxygen and nutrients to the mitochondria for ATP production through a series of mitochondrial respiratory complexes.

Therefore, this current study aimed to primarily investigate the impact of blast injuries with or without HS on mitochondrial structural and functional integrity. We also assess the efficacy of pharmacological interventions, particularly sodium pyruvate, in mitigating mitochondrial dysfunction and improving post-injury outcomes.

### 4.2. Complex I Activity of the Electron Transport Chain in Animals Undergoing High-Intensity Blast Injury +/− HS with Sodium Pyruvate Infusion

**Figure 3:**
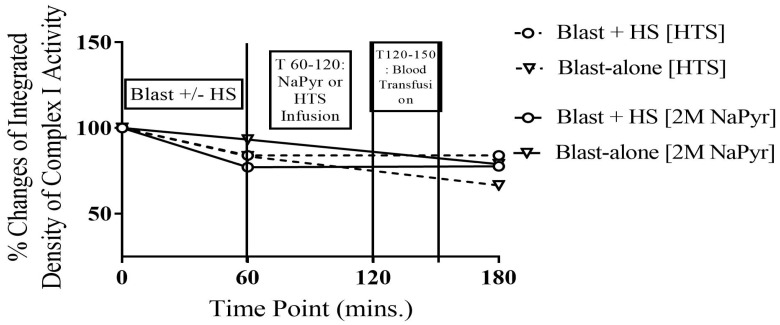
Complex I in whole blood at different time points of 20PSI blast +/− HS injury, and NaPyr treated groups (n = 8) surviving for 4 h. A trend of decreased activity in complex I from the baseline value was observed after the injury (T60), especially more in the 20 PSI-bTBI + HS animals. There were no statistically significant differences between the injury or treatment groups at different time points. The mitochondrial complex I enzyme activity did not deteriorate further after both sodium pyruvate and hypertonic saline infusion. Sham animals did not show any changes; hence, this has not been mentioned here.

Our data demonstrated a significant decrease in the activity of complex I, the largest of the five enzyme super complexes in the mitochondrial electron transport chain following blast injury/HS. As shown in Figure 3, there was a 23% decrease in mitochondrial complex I activity from the baseline value after the combined injury of 20 PSI blast injury and HS. Additionally, we found a 17% decrease in mitochondrial complex I activity in the single injury group of blast alone. The enzyme activity did not further deteriorate after the sodium pyruvate or control hypertonic saline infusion. However, the changes were not statistically significant (*p* > 0.05) between the different injury or infusion groups. For the instrumental and time-matched sham animals receiving no injury or fluid infusion, there were no changes in the complex I activity throughout the experiment.

### 4.3. Complex IV Activity of the Electron Transport Chain in Animals Undergoing High-Intensity Blast Injury +/− HS with Sodium Pyruvate Infusion

**Figure 4:**
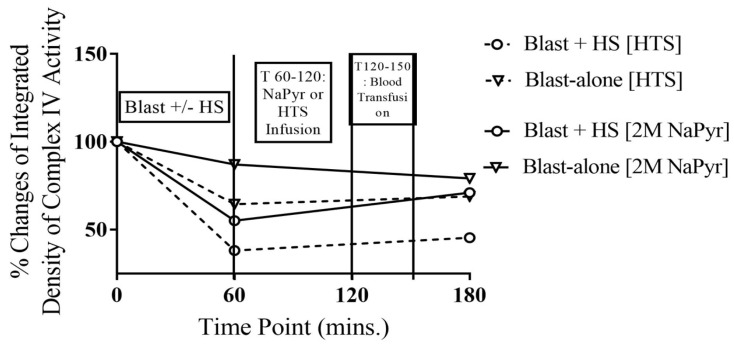
Complex IV in whole blood at different time points of 20PSI blast +/− HS injury, and 2 M NaPyr treated groups (n = 8) surviving for 4 h. A trend of decreased activity in complex IV from the baseline value was observed after the injury (T60), especially more in the 20 PSI-bTBI + HS animals. There were no statistically significant differences between the injury or treatment groups at different time points. Sodium pyruvate or control hypertonic saline infusion slightly improved or stopped further deterioration of the complex IV activity after the blast injury +/− HS.

The animals that survived for 4 h after the high-intensity (20 PSI) blast injury + HS had 45–62% decreased activity in complex IV from the baseline value. In comparison to that, the animals that underwent blast injury showed a 13–36% decrease in complex IV activity, though this is not statistically significant between the groups (Figure 4). Here, we noticed that the degree of mitochondrial damage was again relevant to the severity of injury, i.e., was directly proportional to the injury severity. Sodium pyruvate or control hypertonic saline infusion slightly improved or stopped further deterioration of the complex IV activity after the blast exposure +/− HS. We can conclude that combined blast + HS can induce mitochondrial dysfunction, specifically complex I and IV impairment of the electron transport chain, which may lead to increased free radicals, depletion of cellular ATP, and cell death [11,26,27].

### 4.4. Pyruvate Dehydrogenase (PDH) Activity in Animals Undergoing High-Intensity Blast Injury +/− HS with Sodium Pyruvate Infusion

**Figure 5:**
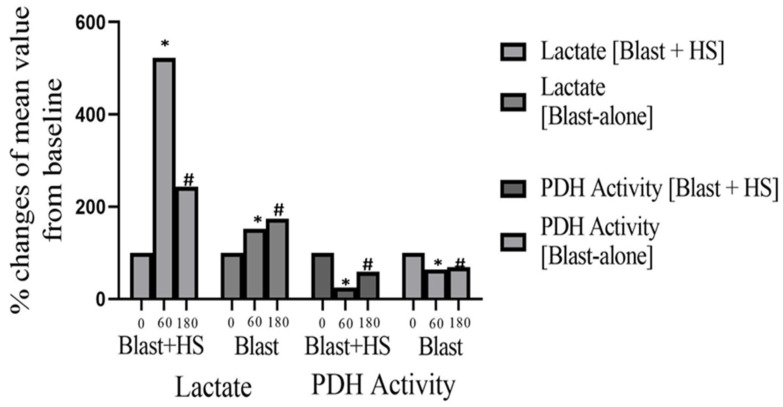
Comparison of plasma lactate and PDH activity at different time points of 20PSI blast +/− HS injury, and 2 M NaPyr treated animals (n = 8) surviving for 4 h. A trend of decreased activity in PDH, and increased concentration of plasma lactate from the baseline value was observed after the injury (T60), especially more and statistically significant (*p* < 0.001) in the 20 PSI-bTBI + HS animals. So, the PDH activity was inversely proportional to the plasma lactate concentration after the injury. Sodium pyruvate infusion had positive effects or improved the situation after the blast injury +/− HS. Symbol “*” denotes significant difference from T0 to T60, and “#” denotes significant difference from T60 to T180.

The pyruvate dehydrogenase complex (PDH) is a crucial mitochondrial enzyme that facilitates the irreversible conversion of pyruvate into acetyl-coenzyme A (CoA) within the mitochondrial matrix. This enzymatic process is pivotal for cellular energy metabolism. PDH is highly susceptible to oxidative stress, making it particularly relevant in the context of our injury model involving blast trauma combined with hemorrhagic shock (HS). As illustrated in Figure 5, PDH activity decreased significantly—by 62–76% from baseline levels—following combined blast and HS injuries. For the blast-alone group, the decline was comparatively less at 36–44%. These reductions in PDH activity correlated with injury severity and were statistically significant (*p* < 0.001). Notably, sodium pyruvate infusion demonstrated greater efficacy in restoring PDH activity compared to control hypertonic saline treatment. 

### 4.5. Plasma Lactate Concentration in Animals Undergoing High-Intensity Blast Injury +/− HS with Sodium Pyruvate Infusion

As discussed earlier, pyruvate dehydrogenase (PDH) displayed significant vulnerability to oxidative damage and subsequent inactivation caused by reactive oxygen species (ROS) due to ischemia following a blast or hemorrhagic shock (HS). PDH, a critical regulatory enzyme within the mitochondrial tricarboxylic acid (TCA) cycle, functions as a pivotal link between aerobic and anaerobic metabolic pathways. Reduced PDH activity in our study emerged as a key contributor to the development of lactic acidosis. Lactic acidosis was observed under conditions of blast and/or HS, wherein oxygen deficiency ranged from partial (hypoxia, ischemia) to severe (hypotension). When comparing the groups, plasma lactate levels were markedly higher in the blast + HS cohort compared to the blast-alone group. This increase in lactate levels after HS was statistically significant (*p* < 0.001), reflecting a five-fold rise relative to baseline values across all blast + HS groups (Figure 5). Although the lactate concentration diminished by the end of the experiments, it did not normalize with either fluid resuscitation approach. The introduction of pyruvate in this experimental model resulted in a notable reduction in plasma lactate levels, suggesting a metabolic shift favoring aerobic respiration facilitated by pyruvate supplementation.

## 5. Discussion

The key results of this study are the reduced plasma mitochondrial enzyme activities of complex I, complex IV, and PDH in response to both blasts alone as well as combined blast + HS injury. The damage to these enzyme activities was more severe in the combined injury compared with blast alone. While pyruvate treatment successfully mitigated the decline in enzyme activities, it failed to fully restore them to baseline levels, indicating the necessity for an integrated therapeutic approach. Our study also revealed an inverse correlation between increased plasma lactate and PDH, demonstrating that PDH can be used as a biomarker for global mitochondrial damage in addition to the lactate as a biomarker of global systemic metabolic failure.

Near infrared spectroscopy is a classical non-invasive technique for hemodynamic monitoring of patients in neuro-intensive care units [28], and can also be used in patients with blast exposure and concomitant hemorrhage. Since organ blood flow cannot be directly monitored in clinical practice, MAP is used, despite limitations, as an estimate of adequacy of tissue perfusion. Due to the similarity of the mean arterial pressure (MAP) at 40 mmHg during HS and the amount of shed blood, throughout the shock period (T60), and during the resuscitation and recovery phases, there were no significant differences in the MAP profile between the pyruvate and HTS treatment groups indicating the possibilities of damaged mitochondrial electron transport chain complexes due to blast exposure and HS combined.

In this study, the animals underwent three repeated blast exposures of 20 PSI at 15 min intervals, thereby mimicking the scenario of soldiers experiencing repeated blast events in combat. The 20 PSI refers to a research study where rats were repeatedly exposed to a blast wave of 20 pounds per square inch (PSI) pressure, simulating a mild traumatic brain injury (TBI) commonly seen in military personnel exposed to explosions. A 20 PSI blast is generally considered a mild TBI, meaning it may not cause significant immediate damage but can lead to subtle neurological changes when repeated [29]. Blast trauma is unique because of its complex mechanism of injury, (1) the rapid change in air pressure can cause hemorrhage and perforation in air filled organs such as lungs, intestine, ears, and concussion, and (2) the flying objects (debris and bomb fragments) can also cause severe hemorrhage in any part of the body resulting in hemorrhagic shock (HS) [3,7]. Therefore, use of a combined injury rat model of blast exposure and HS is highly relevant to our military population and civilians with mass terrorist attacks related to the bomb blasting.

Monitoring of mitochondrial electron transport chain damage using our blood-based dipstick test. Currently, there are no such non-invasive devices to monitor the severity of mitochondrial electron transport chain damage and to correlate them with systemic metabolic parameters such as plasma glucose, lactate, and pyruvate in severely injured non mobile patients. Routine diagnostic tests on metabolic dysfunctions in injured patients have relied mainly on either plasma glucose, lactate, and pyruvate levels measured though handheld i-STAT machines in hospitals. The complicated traditional mitochondrial enzyme assays require purification of mitochondria from tissues obtained by invasive sampling or non-invasive and expensive NMR spectroscopy, which is difficult to use on the battlefield and in mass casualties for metabolic profiling of injury severity [30,31]. It would be preferable for both research and routine clinical use if the severity of mitochondrial damage following blast TBI and/or HS, which are believed to be global in nature (affecting all tissues), could be studied in more easily accessible surrogate tissues, such as saliva and blood. To date, the results of studies on blood have been controversial, due in part to the difficulties in sample collection and processing time for analysis, complex mechanisms of TBI in humans, and associated pre-existing conditions such as PTSD, and mutations in mitochondrial DNA encoding electron transport chain complexes. In addition, variations in animal models of TBI is a major challenge to standardize the biomarker results. In order to provide effective and timely information in the clinic, it will be essential that blood samples could be monitored quickly and repetitively at the point of care with these simple dipstick tests [25]. In this study, we applied the modified proteomic approach using the lateral flow method in blood-based dipstick tests to determine the enzyme activities of mitochondrial complex I, IV, and PDH [14,25]. Importantly, the enzyme activity assays can be run on 50 μL samples (no purification of mitochondria is needed) in 96-well plates. The enzyme activity quantitation assays utilize a binding scheme common to protein sandwich assays using two monoclonal antibodies, enzyme substrate, and a co-factor to determine the enzyme activity. One antibody is embedded in a nitrocellulose membrane of the dipstick while the other is conjugated to gold nanoparticles (Au) in 96-well plates. The integrated density of the protein bands in each sample were measured using Image J. software, version 1.54. The amount of protein signal present on the dipstick is proportional to the amount of target antigen enzyme captured. The results of our blood-based lateral flow immunoassay dipstick test to monitor the severity of combined blast and HS vs. blast alone, and the relation of PDH enzyme activity with plasma lactate, are very encouraging and need to be moved for human studies. Therefore, our first transitional step is to obtain Class III medical device approval from the FDA prior to having it adapted to potential field use. We are planning to test these candidate mitochondrial enzyme activities as biomarkers in brain injury severity and multiorgan failure following HS-related patient population since it is non-invasive. Funding for a clinical trial, and likely for creating an ancillary study of the blood-based dipstick test for mitochondrial biomarker technology in treatment response, will be a combination of DoD and private (i.e., pharmaceutical company) sources.

Mitochondrial complex I is part of mitochondrial inner membrane and the largest respiratory chain complex, composed of about 46 subunits. Complex I dysfunction is associated with various neurodegenerative disorders including Parkinson’s disease as a long-term consequence of TBI [32,33]. Complex I is also the primary route for the oxidation of NADH generated by glycolysis and the citric acid cycle in aerobic cells. Excessive NADH accumulates when complex I activity is deficient [34]. Excessive cellular NADH inhibits glycolysis, the citric acid cycle, and PDH activity, resulting in hyperlactatemia and lactic acidosis, which is a known biomarker of patient’s outcomes following TBI, HS, and resuscitation [35,36]. Complex I is also a known site of reactive oxygen species production in the electron transport chain [37]. The untreated injured tissue cannot compensate for the excessive production of free radicals. Therefore, in the following experiments, we have examined the use of sodium pyruvate to prevent and treat the mitochondrial ETC and PDH damage in TBI [38]. The pharmacological agents such as pyruvate used in this study also improved lactic acidosis which may suggest a mechanism and relationship between oxidative stress and redox imbalance.

In our study, the significantly decreased activity in complex I from the baseline value was observed after the injury (T60), especially more in the combined injury with 20 PSI blast + HS animals. The mitochondrial complex I enzyme activity did not deteriorate further after both sodium pyruvate and hypertonic saline infusion. In addition, pyruvate treatment significantly reduced the plasma lactate levels indicating the protective mechanism of pyruvate through mitochondrial mechanisms in blast exposure as well as in combined injury.

Cytochrome c oxidase or complex IV: Complex IV, located in the inner mitochondrial membrane, serves as the final enzyme in the electron transport chain. This enzyme facilitates the transfer of electrons from four cytochrome c molecules to a single oxygen molecule, resulting in the conversion of oxygen into two molecules of water. As a pivotal component of the electron transport chain, complex IV plays a key regulatory role in oxidative phosphorylation. The enzyme’s function is governed by both nuclear and mitochondrial genes, making it highly susceptible to damage. Any disruption to mitochondrial DNA can significantly impair complex IV activity, leading to a decrease in cellular ATP production with potentially severe consequences. In our study, a significantly decreased activity in complex IV from the baseline value was observed after the injury (T60), especially more in the 20 PSI blast + HS animals compared with blast-alone exposure. Sodium pyruvate or control hypertonic saline infusion slightly improved or stopped further deterioration of the complex IV activity after the blast injury +/− HS.

Pyruvate dehydrogenase complex (PDH) is a gate keeper enzyme for the mitochondria, which decides whether a pyruvate molecule in the cytosol undergoes oxidative phosphorylation for ATP synthesis or anaplerotic activity for repair mechanisms and increased cellular redox status resulting in harmful lactic acidosis [14,19,39,40]. Among the key mitochondrial metabolic enzymes, PDH is very sensitive to oxidative stress [41]. This enzyme plays a pivotal role in the brain’s metabolic processes, as the brain predominantly depends on carbohydrate metabolism for energy. In the context of brain injury, PDH functionality is often impaired, leading to diminished glucose metabolism. This dysfunction contributes significantly to hyperglycemia and lactic acidosis, which are hallmark complications observed in injured neural cells.

Pyruvate has been widely recognized as a metabolic substrate with multiple antioxidant properties and a natural allosteric stimulator of PDH, but its effects on combined blast exposure and HS are entirely unknown. Pyruvate is a monocarboxylate, end product of glycolysis, and it participates in anaplerotic reactions, replenishing the intermediates NAD and NADH within the TCA cycle that can be depleted due to the damaged/over worked mitochondria, thereby maintaining the continuity of the TCA cycle for ATP generation. Pyruvate entry into the mitochondria through the mitochondrial pyruvate carrier (MPC) is also essential for this process, enabling the generation of energy needed for mitochondrial repair mechanisms such as fusion, fission, and mitophagy. In addition, pyruvate has been shown to directly scavenge OH radicals, H_2_O_2_, and peroxynitrite to prevent oxidative and nitrosyl damage to the mitochondrial DNA and its proteins. Mitochondrial function is also closely linked to the calcium handling, and pyruvate metabolism can contribute to maintaining appropriate calcium levels within the mitochondria to prevent its damage [42,43]. Due to these multifaceted properties of pyruvate, it was a novel avenue to examine its protective effects through mitochondrial mechanisms on blast TBI and HS in our study model.

The untreated injured tissue cannot compensate for the excessive production of free radicals. Therefore, we have examined the use of sodium pyruvate to prevent and treat the mitochondrial ETC and PDH damage in TBI [38]. A trend of decreased activity in PDH and increased concentration in plasma lactate from the baseline value was observed after the injury (T60), especially more and statistically significant (*p* < 0.001) in the 20 PSI blast + HS animals compared with animals exposed to blast alone. The lower PDH activity in response to blast +/− HS also resulted in significantly higher plasma lactate concentration after the injury. We have reported that sodium significantly reduced the plasma lactate, which is a reliable biomarker of poor injury outcome in critical care patients. The role of plasma lactate levels as a metabolic biomarker of anerobic respiration, and the severity of brain injury and Glasgow Coma Scale Pupils (GCSP) in TBI patients has been used by clinicians to monitor the unfolding of secondary cell injury [44]. The potential pathways through which pyruvate demonstrates efficacy in mitigating brain injury and preserving pyruvate dehydrogenase (PDH) activity include (1) its role as an antioxidant, which helps neutralize reactive oxygen species (ROS) and minimize oxidative stress, and (2) enhancement of mitochondrial function by serving as a substrate in PDH enzyme reactions and acting as an anaplerotic precursor for the tricarboxylic acid (TCA) cycle, specifically through pyruvate carboxylase. This dual mechanism underscores pyruvate’s therapeutic potential in addressing mitochondrial dysfunction and oxidative damage in injury models.

Carefully looking at our results, we found a relative decline in average plasma PDH activities compared to ETC enzymes (C1, C4) and increased lactate levels in both injury groups (blast vs. blast + HS). A clear dissociation between PDH and key ETC enzymes complexes I and IV levels could be a hallmark of early mitochondrial dysfunction, giving a window of opportunity for mitochondrial-targeted therapies to prevent cellular metabolic failure and organ dysfunction. Different thresholds for oxidative inactivation of various mitochondrial enzymes can explain this variability due to different injury severity (blast vs. combined blast + HS).

### Limitations of the Study

In spite of the exciting results of this study, we had a few limitations. (1) Rat model of blast TBI: In real scenarios, patients are not subjected to anesthesia before a blast. Therefore, they experience the combined effects of an overpressure blast and PTSD-like trauma. While in our model, rats were anesthetized before the blast, and they missed the sound and traumatic experience of blast exposure. (2) Since this was an acute study in our newly designed rat model of combined injury, in future, we plan for long term study using neurological behavioral testing and to try additional drugs combating multiple mechanisms.

## 6. Conclusions

Our findings demonstrate that following blast exposure, with or without severe hemorrhage, mitochondrial key enzymes complex I, IV, and PDH involved in the efficient oxidative phosphorylation process are oxidatively damaged, which may possibly underlie the massive breakdown of the mitochondrial ATP generating system and cause eventual cell death known to occur in our rat model of combined blast and HS injury. The identification of these mitochondrial enzyme activities by our simple, quick, and cost-effective blood-based dipstick test provides new insight into the global mitochondrial mechanisms that take place following blast +/−HS and may provide avenues for possible therapeutic interventions to prevent mitochondrial damage.

The views expressed in this article are those of the authors and do not necessarily reflect the official policy or position of the Uniformed Services University, Department of the Navy, Department of Defense, nor the U.S. Government.

This research was supported by a CDMRP-DM167094 grant to PS.

## Data Availability

All relevant data and information are presented within the manuscript. There are no additional datasets or materials available for sharing beyond what is included in this article.
